# Group independent component analysis of MR spectra

**DOI:** 10.1002/brb3.131

**Published:** 2013-03-13

**Authors:** Ravi Kalyanam, David Boutte, Chuck Gasparovic, Kent E Hutchison, Vince D Calhoun

**Affiliations:** 1 The Mind Research NetworkAlbuquerque, New Mexico; 2 Department of ECE, University of New MexicoAlbuquerque, New Mexico; 3 Department of Neurology, University of New MexicoAlbuquerque, New Mexico; 4 Department of Psychology and Neuroscience, University of ColoradoBoulder, Colorado

**Keywords:** ICA, independent component analysis, LCModel, magnetic resonance spectroscopy, MR spectra decomposition, single voxel spectroscopy

## Abstract

This study investigates the potential of independent component analysis (ICA) to provide a data-driven approach for group level analysis of magnetic resonance (MR) spectra. ICA collectively analyzes data to identify maximally independent components, each of which captures covarying resonances, including those from different metabolic sources. A comparative evaluation of the ICA approach with the more established LCModel method in analyzing two different noise-free, artifact-free, simulated data sets of known compositions is presented. The results from such ideal simulations demonstrate the ability of data-driven ICA to decompose data and accurately extract components resembling modeled basis spectra from both data sets, whereas the LCModel results suffer when the underlying model deviates from assumptions, thus highlighting the sensitivity of model-based approaches to modeling inaccuracies. Analyses with simulated data show that independent component weights are good estimates of concentrations, even of metabolites with low intensity singlet peaks, such as scyllo-inositol. ICA is also applied to single voxel spectra from 193 subjects, without correcting for baseline variations, line-width broadening or noise. The results provide evidence that, despite the presence of confounding artifacts, ICA can be used to analyze in vivo spectra and extract resonances of interest. ICA is a promising technique for decomposing MR spectral data into components resembling metabolite resonances, and therefore has the potential to provide a data-driven alternative to the use of metabolite concentrations derived from curve-fitting individual spectra in making group comparisons.

## Introduction

Proton magnetic resonance spectroscopy (^1^H-MRS) is a powerful, noninvasive method that allows in vivo estimation of metabolite concentrations in a tissue volume. It has enabled extensive investigation and characterization of biochemical profiles in a variety of healthy and pathological tissues. Many neurological studies have shown the importance of ^1^H-MRS in diagnosis, treatment monitoring, and prognosis of major diseases including Alzheimer's, cancer, dementia, and multiple sclerosis (Jansen et al. 2006). Significant and sustained research has been conducted over the years using ^1^H-MRS in an effort to fulfill its potential as a clinical tool.

A typical in vivo brain ^1^H-MRS spectrum consists of resonances from metabolites of interest along with features such as residual water signal, baseline fluctuations, and other artifacts not of interest. A common approach to making meaningful comparisons across subjects, brain regions, or pathologies involves quantifying metabolites in terms of concentrations. Popular methods such as LCModel (Provencher 1993), a frequency-domain approach, or JMRUI, a time-domain approach (Naressi et al. 2001), fit a model function derived from an in vitro or simulated set of metabolite profiles to data. Both time- and frequency-domain quantification approaches employ a variety of data preprocessing techniques to remove or model confounding features in order to improve estimation accuracy (Helms 2008) and often allow semiautomated processing of data to produce consistent quantitation, without special expertise. While model-based approaches bring the ability to resolve overlapping resonances, the sensitivity of their estimates to modeling inaccuracies is a serious concern and making an appropriate choice of model spectra is essential (Kreis 2004).

In this article, we present a data-driven approach for group level analysis of MR spectra based on independent component analysis (ICA). This approach is applied collectively to all analyzed spectra as a group, and resolves individual spectra into linear combinations of a set of components maximally independent of each other. Unlike model-based approaches, ICA makes no assumptions on underlying source distributions, and therefore, can be relatively robust when applied to diverse data sets. In addition, ICA can potentially extract coherent variations between resonances from the whole spectra, which may be useful in identifying metabolites that covary. Furthermore, features of the spectra that are generally not of interest, such as line broadening and baseline fluctuations, can often be resolved into separate components, allowing the resonances of interest to be quantified without the potential confound of these artifacts.

A statistical technique that has been used for multivariate analysis of spectroscopy data is the model-independent principal component analysis (PCA) (Stoyanova and Brown 2001). ICA is a conceptually similar technique that has been widely used in functional magnetic resonance imaging analysis (Calhoun et al. 2003, 2009) and has been shown to model individual subject variations well (Allen et al. 2012). It has also been used in few prior studies to resolve ^1^H-MR spectra and extract independent components (ICs) that could separate pathologic tissues (Ladroue et al. 2003; Pulkkinen et al. 2005). Both of those studies demonstrated, using fast ICA (Hyvarinen 1999), that components maximizing independence can group resonances effectively to classify healthy brain tissue and grades of tumor tissue. Additionally, a few simulation studies examining the effects of line broadening and noise on the extracted components have also been published (Ladroue et al. 2003; Hao et al. 2009).

However, no previous published study directly compared PCA or ICA results with more established methods, such as LCModel, which could present a more convincing case for the use of ICA in MR spectral analysis. In this article, we present comparative evaluations of ICA and LCModel in analyzing two simulated data sets, each composed of metabolites typically found in human brain, but generated using different sets of basis spectra. Though LCModel has been compared to other model-based methods (Hofmann et al. 2002; Kanowski et al. 2004), to our knowledge, the present study is the first to compare the model-based LCModel with the model-independent ICA.

Simulation results highlight the sensitivities of model-based approaches to modeling inaccuracies and the advantages of a data-driven approach in this respect. Further, we demonstrate that the components extracted based on independence criterion alone are good approximations of the underlying basis spectra and that the component weights can be used instead of concentration estimates as parameters in comparing spectra. Finally, we also apply ICA analysis to an in vivo single voxel data set of 193 spectra and compare components and component weights to the basis spectra and concentration estimates from LCModel analyses. We show that ICA component weights and LCModel results correlate to different degrees depending on the metabolite. ICA is also able to capture low intensity singlet peak signals such as those that may arise from scyllo-inositol (s-Ins). Overall, our results show that the data-driven ICA could be a valuable tool for group analysis of MR spectra.

## Materials and Methods

ICA is a linear time-invariant method that decomposes a set of observations into a linear combination of basis signals. It may be seen as a higher order generalization (Comon 1994) of PCA, often employed for dimension reduction prior to ICA. Unlike PCA, which imposes independence up to second order and defines orthogonal directions, ICA minimizes statistical dependence between its components, and is uniquely defined when at most one component is Gaussian (Bell and Sejnowski 1995). As MR spectra are made of contributions from individual metabolite spectra that can vary independently, estimated ICs are expected to characterize well any independently varying signals from metabolites.

The linear construct in equation (2) expresses a composite spectrum or observation **x**_***n***_, as a linear combination of a set of *k* components or sources **s**_***i***_, weighted by mixing coefficients ***a***_***i***_.


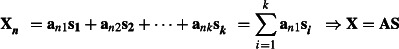
(1)

ICA estimates the matrix **W** that demixes multivariate data **X** to extract estimates **Y** of sources **S**, such that **Y = WX** are mutually independent. If the sources are mutually independent, then **Y** is close to **S** and **W** is the pseudoinverse of **A**. A variety of algorithms implementing the iterative learning and estimations of **W** exist. They construct unmixing matrix **W** such that negentropy, or distance from normality, of **Y** is maximized. As negentropy is difficult to compute, many algorithms rely on kurtosis as its estimate. In our implementation, we use the infomax algorithm (Bell and Sejnowski 1995) on the real part of input spectral data from our simulation experiment or in vivo and demonstrate ICA's ability to resolve spectra and extract resonances having different statistical properties.

### Data simulation

The design objective of our simulation experiment was to assess how well ICA extracts underlying components and ground truth-mixing coefficients from simulated data resembling in vivo human brain MR spectra; and to explore how ICA results compare to LCModel results from the same data. Data simulated with two different sets of modeled resonances, with no added noise or artifacts, provided a means to compare ICA approach with LCModel analysis, as well as to establish upper bounds of ICA's ability in MR spectral applications.

The composition of our basis set of metabolites was based on a list of metabolites typically included in a LCModel basis set with analysis window of 1.8–4.2 ppm, the analysis window used in a prior report on these data (Yeo et al. 2013). The basis set was composed of 12 metabolites: aspartate (Asp), creatine (Cr), γ-amino butyric acid (GABA), glucose (Glc), glutamine (Gln), glutamate (Glu), *N*-acetyl aspartate (NAA), the *N*-acetyl peak of *N*-acetylaspartylglutamate (NAAG), the trimethyl amine group of phosphocholine (PCh), taurine (Tau), and myo-inositol (m-Ins) and its isomer scyllo-inositol (s-Ins).

For each of the metabolites in our basis set, we obtained two sets of modeled resonances with matching experimental parameters: a point resolved spectroscopy (PRESS) sequence acquired at 3T with a 40-msec echo time and 1600-Hz bandwidth. The first set of spectra, called here LCModel basis, was generated from the LCModel basis set provided by the developer of LCModel. The spectra in this basis set were resampled to match the resolution and bandwidth of in vivo spectra and saved in a matrix of length 512. The second set of spectra, called here GAVA basis, was simulated using a predefined library of pulse sequences in GAVA (Soher et al. 2007), a user friendly front end for the GAMMA MRS simulation libraries; the 1024 data point timed-domain model data were converted into spectral domain using the discrete fast fourier transform (FFT) and saved in a matrix of the same dimensions as LCModel basis. We omitted Glc from GAVA basis set, but replaced it with Glycine (Gly), which was not part of the LCModel basis set we used to analyze the data.

In order to closely mimic in vivo spectra, we used concentration estimates from LCModel analysis of in vivo data as ground truth-mixing coefficients. For Cr, we used combined estimates of Cr and phosphocreatine (PCr) as the reference; likewise, we used combined estimates of PCh and glyco-phosphocholine (GPC) as the reference for PCh. For Gly in the GAVA basis, which LCModel does not use, we used concentration estimates of Glc, present in normal adult human brain at levels comparable to Gly (∼1 mmol/kg) (Govindaraju et al. 2000). We obtain 193 sets of mixing coefficients from LCModel analysis of in vivo data.

Each composite spectrum was generated by linearly mixing a chosen set of basis spectra, weighted by any one set of mixing coefficients. Using the entire set of mixing coefficients, two sets of 193 simulated spectral data were generated: one using LCModel basis and the other using GAVA basis. Such simulated data can be directly analyzed by ICA, but for use with LCModel, each composite spectrum was converted into 1024 data point complex time-domain data using inverse FFT and stored in individual files.

### In vivo acquisition

MR data were collected from 141 male, 90 female subjects (*N* = 231), aged between 18 and 56, with a median age of 30, enrolled in three substance abuse studies at the Mind Research Network, conducted in accordance with protocols approved by the human research review committee of the University of New Mexico. Subjects, none of whom are controls, provided informed consent prior to their admission to the studies, and were compensated for their participation. None of the participants were taking psychoactive medications, or had any history of a substance dependence disorder other than alcohol or tobacco dependence in the 6 months preceding enrollment.

All spectroscopic and image data were acquired on a Siemens (Erlangen, Germany) TimTrio 3T scanner equipped with 40 mT/m gradients, body coil, and 12-channel receive-only phased array head coil. *T*1-weighted structural images acquired with a single excitation 5-echo magnetization prepared rapid gradient echo sequence with TE = [1.64, 3.5, 5.36, 7.22, 9.08] msec, TR = 2.53 sec, *T*1 = 1.2 sec, flip angle = 7°, slice thickness = 1 mm and resolution = 256 × 256 mm^2^ was used to prescribe a single 12 cc (20 × 20 × 30 mm^3^) ^1^H-MRS voxel in the anterior cingulate region of the brain. Data were collected with body coil excitation, in conjunction with head matrix coils in receive mode, using a PRESS sequence with TR/TE = 1.5 sec/40 msec, 1600-Hz bandwidth and 192 averages. Scanner preprocessing software corrects zero-order phase differences before combining individual spectra from different channels (Natt et al. 2005), averages acquisitions from multiple scans, and saves acquired data in 1024 complex time-domain data points. For use with LCModel, a water spectrum with 16 averages was also acquired from the same voxel.

In the ICA analysis, we used water-suppressed data, which had been normalized by the scanner software using a single scan water reference acquisition (Natt et al. 2005). As ICA works collectively on all spectra, data from all subjects were read and stored in a matrix. Also, as our ICA approach requires complex, frequency-domain data, the acquired complex time-domain data were converted into spectral domain using FFT.

In vivo spectra were corrected for B_0_ variation by using real part of the *N*-acetyl peak of NAA spectrum from LCModel basis to align spectra. Following spectral alignment, we sought to exclude spectra that could unduly bias component estimation and extraction. Spectra with suspect LCModel results, such as those with large full-width half-maximum (FWHM > 0.072 ppm) or poor signal-to-noise ratio (SNR < 15) or simply a bad fit were excluded. We also excluded spectra if the associated LCModel concentration estimates of any metabolite were more than 3.5 standard deviations from the corresponding mean. Finally, we applied an objective data-driven quality control that excluded any spectrum with any data point in the analysis window more than 3.5 standard deviations from corresponding point in the mean spectrum (generated from all included spectra). Though arbitrary, such a choice allowed us to exclude very few poor quality spectra and realize an in vivo data set with no variance outliers (*N* = 193).

### ICA analysis

ICA was performed over the same analysis window used in the LCModel analysis (1.8–4.2 ppm), using the real part of the spectra. Such an approach is suitable for the linear unmixing problem in ICA and also suits the infomax algorithm, which works well with real valued data. Without any further preprocessing, the spectra were mean centered (demeaned) and factorized using singular value decomposition to perform PCA. The number of retained principal components was determined using minimum description length criteria (Rissanen 1983; Ojanen et al. 2004) along with a priori information on the data matrix, and the expected number of biochemically interpretable components. We carried out multiple (10) ICA runs to check for spurious or false convergences and observed that, in each run, ICA converged in 1–2 min to a consistent set of components, suggesting a single run was enough. All the components were normalized and sign corrected if necessary, to resolve a permutation ambiguity that arises with ICA. The components from different runs were then clustered into groups based on correlation distances and cluster centroids were used as ICs in further analysis; corresponding component weights were extracted by projecting the components onto the data. In contrast to the LCModel estimates, which are quantifications of concentrations of individual metabolites in the basis set, ICA estimates are the weights associated with the independent resonances, which may correspond to metabolite resonances and can capture ground-truth concentrations accurately. Hereafter, the terms ICA estimates and component weights will be used interchangeably.

The extracted ICs were compared with the underlying basis spectra, to identify and associate components with modeled resonances. Each component was automatically paired with a basis spectrum based on their similarity, as measured by the Pearson product-moment correlation coefficient (*r*), called spectral correlation of the matched pair. We also calculated a weights correlation, measured by Pearson correlation coefficient of the component weights with the ground truth-mixing coefficients. For in vivo data, due the absence of absolute references, we used LCModel basis to match and identify components, and used LCModel concentration estimates as a form of ground-truth reference.

### LCModel analysis

LCModel analysis was carried out with no explicit eddy-current compensation within a 1.8–4.2 ppm analysis window, which results in automatic exclusion of alanine, macromolecules, and lipids from the basis set. LCModel fits each individual spectrum using the remaining resonances in the window. For in vivo analysis, we use all those resonances, but for both simulation analyses, we omitted negative creatine CH_2_ singlet (-CrCH_2_) and guanidinoacetate (Gua) from the basis. This ensures LCModel is posed the simpler problem of fitting the data with the known composition. Also, while our in vivo analysis used the acquired water spectrum as internal water reference to estimate absolute concentrations, our simulated data estimates were normalized by the Cr + PCr intensity.

### Additional analyses

We closely examined how ICA resolves our basis set containing a mix of weak and strong metabolites having a wide array of resonances, all of which are not necessarily mutually independent. In particular, we investigated the effect of setting the number of extracted ICs to a number different than the number of basis spectra underlying our simulated data. As previously, the real part of the GAVA-simulated spectra within the analysis window was demeaned and dimension reduced. Though the simulated data were generated with 12 GAVA basis spectra, the number of components retained successively varied from six to 18 and each time ICA was performed as previously and the extracted ICs were matched with basis spectra; spectral and weights correlations were also estimated each time.

We also carried out some analysis to examine how well the ICA estimates could capture a certain relationship between ground truth and an attribute of interest (phenotype). Toward this effort, we generated a set of pseudorandom vectors, each correlating with ground truth, with a preset correlation score (*r* = 0.5). Each vector mimics a neurological, physiological, or physical attribute correlated with ground truth. We then computed how those vectors correlated with the ICA estimates. By generating multiple (*N* = 100) realizations of such vectors, and computing their correlations with the ICA estimates each time, we observed how accurately could the ICA estimates capture the relationship between “phenotypes” and ground truth.

As the ICs from in vivo analysis do not have a ground truth, we sought to show that the select ICs indeed originated from metabolic sources, and not from confounds or nuisance artifacts associated with real data. To this end, we examined how the fractional tissue volumes in the spectroscopic voxel correlated with LCModel estimates or component weights. As cerebrospinal fluid (CSF) is mostly void of observable metabolites (Gasparovic et al. 2006), when the fractional tissue volume is high, more metabolites exist in the spectroscopic voxel and therefore both the estimates are expected to correlate positively with the fractional tissue volume. However, this effect is expected to disappear when the estimates are normalized with a reference metabolite estimate from within the voxel. The fractional tissue volumes in the spectroscopic voxel were estimated by segmenting high-resolution *T*1-weighted images into gray matter, white matter, and CSF using the unified segmentation approach available in SPM5 (Ashburner and Friston 2005) and averaging fractional tissue volumes of the *T*1-pixels within the spectroscopic voxel.

## Results

**Figure 1:**
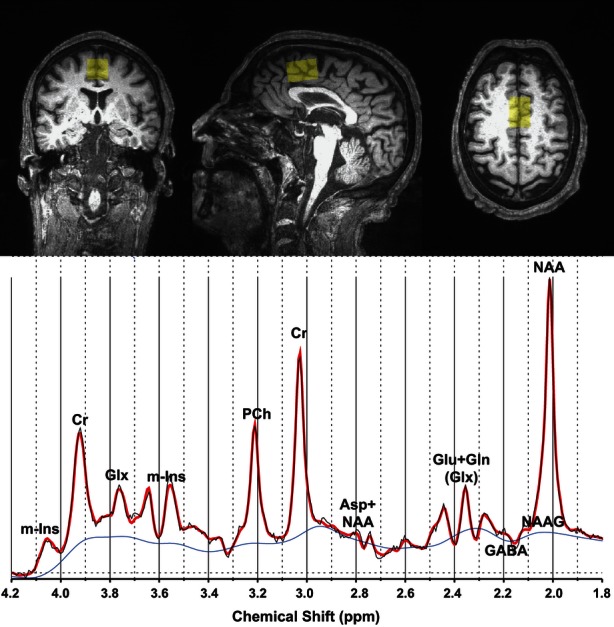
Location of the voxel and typical MR spectra: 12 cc spectroscopic voxel is positioned in the anterior cingulate region. LCModel plot of a typical in vivo metabolite spectrum in 1.8–4.2 ppm analysis window shows the real part of the referenced and phase-corrected spectrum in faint grey line; smooth red line is the LCModel fit, and the blue line is the estimated baseline. Some resonances are identified with associated metabolites (Glx refers to Glu + Gln). MR, magnetic resonance; Glu, glutamate; Gln, glutamine.

The location of the spectroscopic voxel in vivo experiments, in the anterior cingulate region of the brain, is shown in Figure 1. Also shown is the LCModel output that presents a typical metabolite spectrum and LCModel's fit to the spectrum; some key resonances are labeled and the estimated spectral baseline is also shown.

### Simulation

As the composition of our simulated data is known, we only extracted as many ICs as the number of sources (12) underlying the data; ICs were paired with basis spectra, and corresponding component weights were also estimated. Table 1 presents spectral correlations between the real parts of LCModel and GAVA model spectra; notice the correlations are not the same across metabolites. For each set of simulated data, the table also captures spectral and weights correlations and the correlations of LCModel estimates with the ground truth. Note the high spectral and weights correlations revealing the ability of ICA to resolve MR spectra and extract ICs substantially resembling underlying basis spectra. Also notice LCModel results are strongly predicated upon the basis spectra underlying the simulated data.

**Table 1:** Results from 193 spectra simulation experiments: Results from simulated data generated with LCModel and GAVA basis shown. Notice the spectral correlations between the modeled spectra are not the same across metabolites. Both the LCModel and ICA estimates (weights) correlate well with ground truth when analyzing data simulated with LCModel basis spectra. LCModel estimates suffer when analyzing data simulated with GAVA basis spectra, as the data deviate from the assumed model, whereas ICA results remain strong

	Asp	Cr	GABA	Glc/Gly	Gln	Glu	m-Ins	NAA	NAAG	PCh	s-Ins	Tau
Gava-LCModel spectral corr.	0.072	0.887	0.907	–	0.792	0.557	0.764	0.732	0.883	0.1	0.801	0.586
Truth-LCModel conc.^1^	0.999	0.998	0.995	0.999	1	0.999	0.999	0.999	0.999	0.997	0.999	0.998
LCModel-ICA spectral corr.^1^	0.995	1	0.983	0.947	0.978	0.986	0.99	1	0.999	1	1	0.997
Truth-ICA weights^1^	0.996	1	0.994	0.982	0.997	0.988	0.999	1	0.997	0.998	0.998	0.929
Truth-LCModel conc.^2^	0.407	0.994	0.886	–	0.994	0.984	0.869	0.99	0.586	0.343	0.988	0.966
Gava-ICA spectral corr.^2^	0.999	1	0.989	1	0.989	0.983	0.869	0.999	0.999	1	1	0.994
Truth-ICA weights^2^	0.999	1	0.971	0.959	0.993	0.996	0.999	1	0.931	0.996	0.993	1

ICA, independent component analysis; Asp, aspartate; Cr, creatine; GABA, γ-amino butyric acid; Glc, glucose; Gln, glutamine; Glu, glutamate; m-Ins, myo-inositol; NAA, *N*-acetyl aspartate; NAAG, *N*-acetyl peak of *N*-acetylaspartylglutamate; PCh, trimethyl amine group of phosphocholine; s-Ins, isomer scyllo-inositol; Tau, taurine.

1 Using LCModel basis set.

2 Using GAVA basis set.

**Figure 2:**
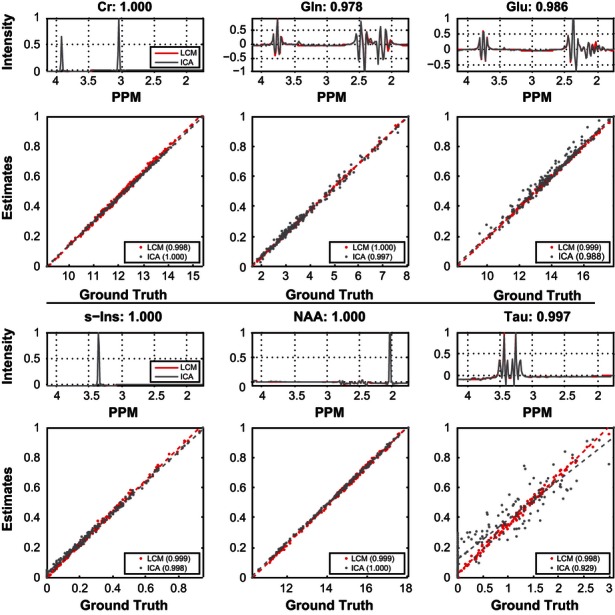
Results from simulated data generated with LCModel spectra: Real part of select LCModel basis spectra and the matching ICs, both zero-mean, unit-norm shown; PPM scale is presented for reference only. Also shown are scatter plots of corresponding estimates, LCModel concentration and independent component analysis (ICA) weights, both normalized on a zero-to-one scale, plotted against ground-truth mixing coefficients. Least squares fit lines for the scatters and Pearson correlation scores for the spectra and scatters are also shown. See good overlap between the basis spectra and matching ICs, across board. The near-perfect LCModel scatter is in line with expectations, because the basis spectra underlying the data are LCModel's own; tight scatter of ICA weights shows ICA does comparable job, without assuming underlying basis distributions.

The results from analysis of the data generated using LCModel basis are shown in Figure 2. The figure shows the real part of the spectra of select metabolites, superimposed with matching ICs; spectra are demeaned and intensity normalized (zero-mean, unit-norm). Shown below the spectra are the scatter plots of the estimates from ICA and LCModel plotted against ground truth-mixing coefficients. The estimates are normalized on a scale of 0 to 1 and least squares fit lines for the scatters are also shown. Note the high spectral correlations across the board and the near-perfect overlaps between the ICs and basis spectra. Also notice how well LCModel resolves Glu, Gln from simulated data, which is not common in the in vivo case. The tight scatter of LCModel estimates are not surprising, given LCModel's own basis spectra were used to generate the data in the first place. This shows that LCModel estimates are accurate when modeling assumptions are valid, and also validates our simulation experiment. Notice that the scatter in ICA estimates is also comparably small, with high correlation scores.

**Figure 3:**
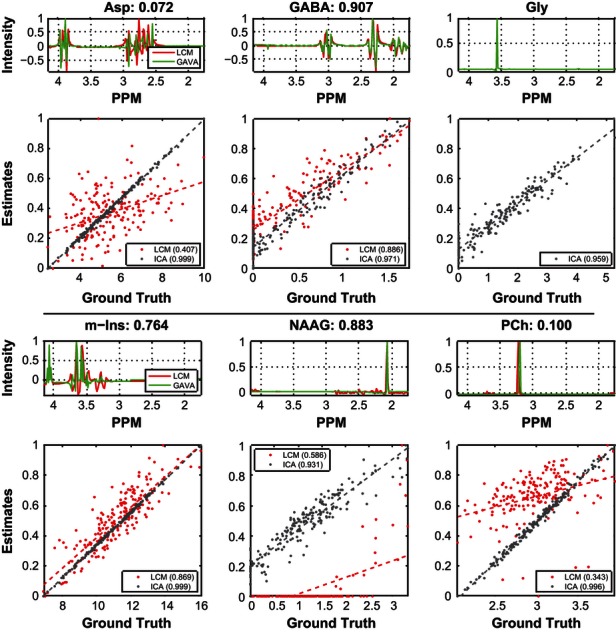
Results from simulated data generated with GAVA spectra: Real part of select LCModel basis spectra and matching GAVA basis spectra, both zero-mean, unit-norm shown; extracted ICs that closely resemble GAVA basis, not shown; PPM scale is presented for reference only. Also shown are scatter plots of LCModel concentration and ICA weights, both normalized on a zero-to-one scale, plotted against ground-truth mixing coefficients; least squares fit lines and Pearson correlation scores for the scatters are also shown. Non-overlap of the spectra reveal differences exist between the two models, and poor LCModel scatter is a direct consequence of such modeling differences; also note LCModel does not include Gly basis or output its estimates. The tight scatter of ICA weights shows that ICA, being data-driven, is robust to the differences in underlying spectral properties. ICA, independent component analysis; Gly, glycine.

Figure 3 captures the results from analysis of the data generated using GAVA basis. The real part of select LCModel and GAVA basis spectra, all with zero-mean and unit-norm are shown; extracted ICs closely resembling GAVA basis are not shown. Although both the models were simulated with similar sequence parameters and experimental conditions and show great similarities, their spectral patterns are not identical and the occasional lack of overlap reveals that differences exist between the models, as captured by their spectral correlations. Notice the correlations of LCModel estimates against ground truth-mixing coefficients are considerably weak when the basis spectral correlations are weak, revealing that LCModel estimates suffer when the data are not consistent with the modeling assumptions. In contrast, the ICA estimates are highly accurate and underscore the robustness of ICA to the changes in spectral profiles underlying the data. Also note we could not provide the scatter plot for Gly estimates from LCModel, as Gly was not part of our LCModel basis set.

**Figure 4:**
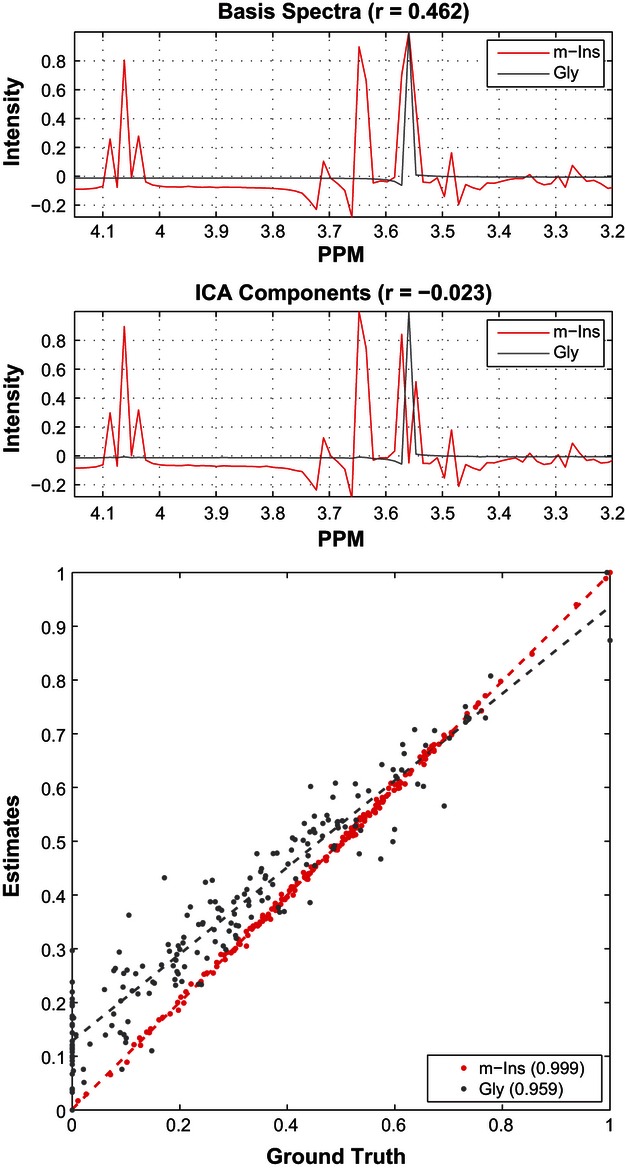
Effects of Independence on extracted components: Real part of GAVA basis spectra of Gly and m-Ins, and corresponding ICs shown; plotted spectra are zero-mean, unit-norm and PPM scale is presented for reference only. While modeled resonances of both metabolites have a common peak at ∼3.56 ppm, ICA resolves this peak as Gly singlet; as ICA enforces independence by minimizing mutual information between ICs, the peak disappears from the m-Ins like component. Though spectral correlation of m-Ins is slightly lower (see Table 1), its estimate is accurate and even better than that of Gly. ICA, independent component analysis; Gly, glycine; m-Ins, myo-inositol.

Figure 4 shows zero-mean, unit-norm modeled resonances of m-Ins and Gly, which are correlated due to the peak at 3.56 ppm (*r*∼0.46). Also shown are the two matching ICs, which are decorrelated, because ICA, as expected, fully resolves the 3.56-ppm peak separately, as Gly. Though the missing spectral peak in the m-Ins resonance results in slightly lower spectral correlations (see Table 1), the weights estimation was not compromised; in fact, the more accurately extracted Gly resonance has comparatively larger scatter.

**Figure 5:**
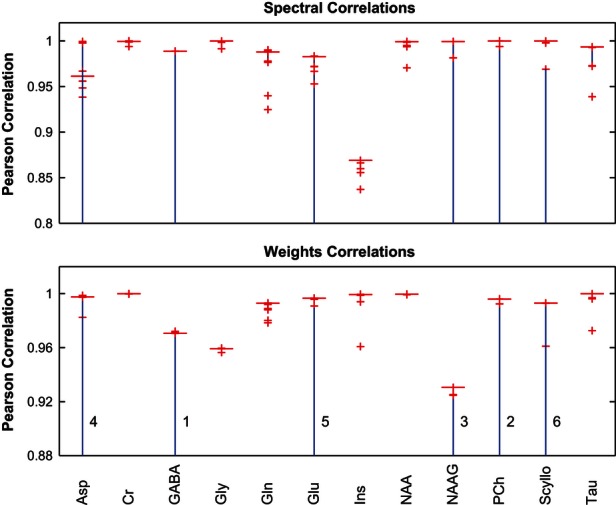
Impact of number of ICs on correlation scores: Results from independent component analysis (ICA) analysis of simulated data generated with 12 components GAVA basis spectra when the number of ICs extracted from were varied from 6 to 18 shown. In these compact box plots, each cross-line is a correlation score, and the medians are marked by broad lines. High spectral and weights correlations show minimal impact of the number of components on the extracted ICs. ICs that do not get resolved when fewer than 12 components are extracted are identified with drop-down lines and their order of disappearance is also shown.

Figure 5 shows spectral and weights correlations when the number of ICs extracted from data set simulated with 12 GAVA basis spectra is varied from 6 to 18. The illustration combines compact box plot and scatter plots; each correlation score is a cross line, and medians are marked by broader lines. Notice the high spectral and weights correlations, showing little effect of the number of ICs on the resolved components. When fewer than 12 ICs were extracted, few components will not get resolved. Some ICs are more readily resolved than others and the ICs that do not get resolved or disappear are identified with the drop-down lines and the adjacent numbers show their order of disappearance.

**Figure 6:**
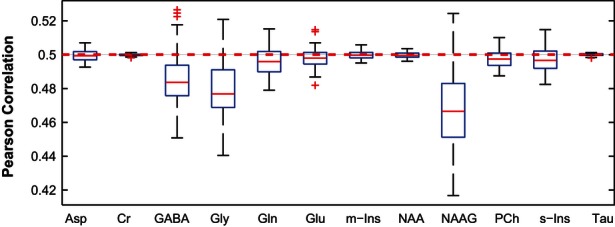
Phenotypes correlations: Box plots show correlations of independent component analysis (ICA) estimates (weights) with multiple realizations of phenotypes matrix, each column of which is correlated to ground-truth at *r* = 0.5. The spread of the boxes correspond to dispersion of ICA estimates. Higher the correlations between ICA estimates and ground-truths, smaller the dispersion and size of the box; phenotypes correlations of with ICA estimates barely inflate ground-truth correlations, and are about the same significance, in most cases.

The box plots in Figure 6 show the results from phenotypes simulation. The boxes represent the middle quartiles (between 25th and 75th percentiles) of the correlation scores between ICA weights and phenotypes matrix realizations. The size of the box corresponds to the dispersion in the estimation of ICA weights; notice the variability in the scatter plots in Figure 3 directly corresponds to the size of the corresponding boxes. Except for GABA, Gly, and NAAG, the correlations are virtually no different from the ground-truth correlations set at *r* = 0.5. Even in the case of the worst performing metabolite, the weights show a correlation with *r* ∼0.42, only slightly lower.

### In vivo application

Similar to the procedure used with simulated data, in vivo spectral data (*N* = 193) were demeaned and rank reduced using singular value decomposition, to 20 components, before multirun ICA. The extracted ICs were automatically paired with LCModel basis and corresponding weights were also estimated. Table 2 lists those select pairs with significant spectral correlations, and captures both spectral and weights correlations. While ICs resembling the m-Ins signal and the singlet resonances of NAA, NAAG, Cr, PCh, and s-Ins were readily identified, no ICs resembling resonances from Asp, Glu, Gln, and GABA were discerned. The table also captures how the ICA and LCModel estimates relate to the fractional tissue volume in the spectroscopic voxel. More the tissue fraction, more signal is detected and the estimates are larger. Therefore, without normalization, the estimates show similar, positive correlations with tissue volumes; the correlations are weak possibly due to the lack of perfect spatial overlap between metabolite and water volumes. However, when normalized neither set of estimates correlates with tissue volumes, as expected.

**Table 2:** Results from ICA analysis of 193 spectra in vivo data: Components identified based on spectral correlation with matching LCModel spectra are shown. The correlations between the LCModel and ICA estimates (weights), both NAA normalized, are appreciable given both are estimates of some unknown ground truth. Also shown are the correlations of the estimates with the fractional tissue volume in the spectroscopic voxel; while the estimates correlate positively with the tissue volume without normalization, they are decorrelated when NAA normalized

	Cr	m-Ins	NAA	NAAG	PCh	s-Ins
Spectral correlations	0.794	0.743	0.949	0.726	0.836	0.731
Weights*: LCModel conc.*	0.826	0.677	–	0.261	0.879	0.632
Weights*: Tissue volume corr.	0.021	0.002	–	−0.175	−0.037	0.158
LCModel conc.*: Tissue volume corr.	−0.1	0.047	–	0.027	−0.197	−0.199
Weights: Tissue volume corr.	0.531	0.468	0.448	0.292	0.408	−0.009
LCModel conc.: Tissue volume corr.	0.521	0.423	0.522	0.129	0.213	−0.114

ICA, independent component analysis; NAA, *N*-acetyl aspartate; Cr, creatine; m-Ins, myo-inositol; NAAG, *N*-acetyl peak of *N*-acetylaspartylglutamate; PCh, trimethyl amine group of phosphocholine; s-Ins, isomer scyllo-inositol.

* Normalized with NAA estimates.

**Figure 7:**
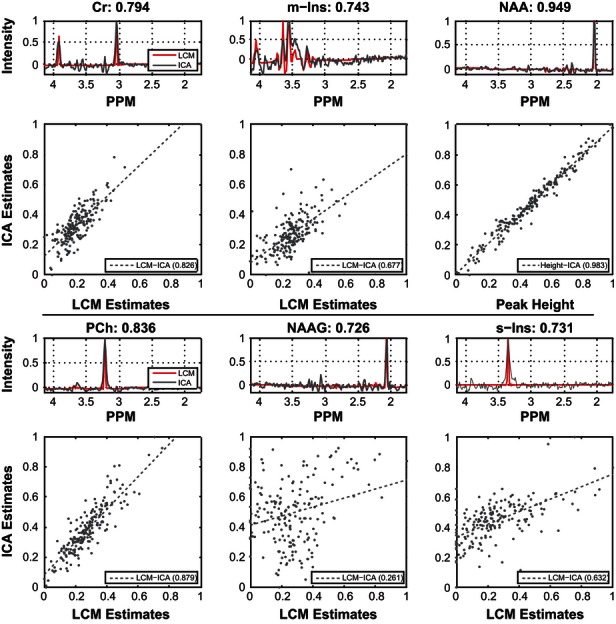
Results from 193 spectra in vivo data: Select ICs and matching LCModel spectra shown; all spectra zero-mean, unit-norm. See resonance peaks of the ICs substantially overlapping matched basis spectra; also notice minor covarying resonances along the baseline. Due to lack of ground-truths for in vivo, we show scatter plots of the estimates, both NAA-normalized, plotted against each other, along with least squares fit lines. Given the complexities in the real data and limitations of either approach, the dispersion in the scatters is hardly surprising. However, note the scatter of ICA estimates (weights) of NAA against the peakheight of the spectral input to ICA is comparatively tighter, as expected. NAA, *N*-acetyl aspartate; ICA, independent component analysis.

Figure 7 shows results from ICA analysis of in vivo data, in the absence any ground truth, plotted against LCModel references. The components with significant spectral correlations are overlaid on the matching real part of the paired LCModel basis spectrum; spectra plotted are demeaned and intensity normalized. Notice the components substantially overlap paired basis spectra at the major peaks, with some differences apparent around the baseline, attributed to covarying resonances; for example, the peaks around 2.4 ppm of NAA-like component seem to arise from Glu, based on Pearson correlation in the spectral subspace (*r* = 0.612). Resonances such as those from Asp, GABA, or Gln are not readily discerned from in vivo data and therefore not presented. Also shown below each set of spectra are the scatter plots of the ICA estimates (weights), plotted against LCModel estimates, both expressed as a ratio with NAA; least squares fit lines for the scatter plots are also shown. As NAA is the reference metabolite, its scatter plot is not constructed; instead, we present a scatter plot between the weights of NAA component and the peak value of the spectral input to ICA. The strong linearity in that relationship shows that the peak value of the spectra, determined by its dominant peak (NAA), linearly relates to the area under the NAA peak (weight).

**Figure 8:**
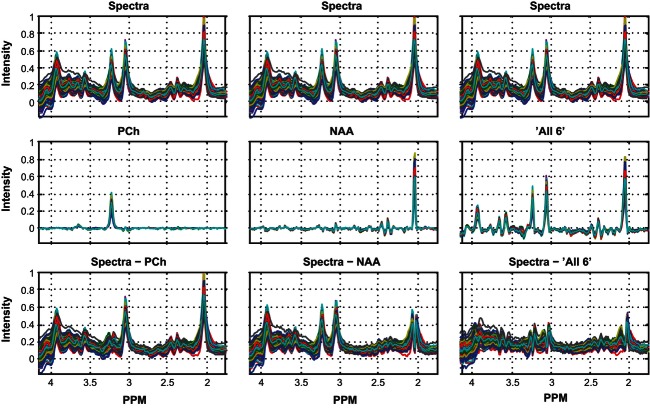
Cut-out plots from in vivo experiment: Top row shows the input to ICA, real part of in vivo spectra from 193 subjects. Mid row captures the variability explained by select component(s): Cho, NAA, and “all select spectra”. Bottom row captures the variability unexplained by the component(s) immediately above. NAA, *N*-acetyl aspartate; ICA, independent component analysis.

Figure 8 illustrates the capability of ICA to extract certain resonances of interest in the presence of confounds, and toward this, we present three sets of plots in columns. The first plot in each set (top row) is the 193 subjects spectral data input to ICA, the composite spectra reconstructed from principal components. The second plot (middle row) is the variability in the data explained by an individual independent component or group of ICs. The final plot (bottom row) is the residue or the variability unexplained by the respective component(s). For the purposes of this illustration, we selected two individual ICs (Cho, NAA), and the whole set of six ICs (Cr, m-Ins, NAA, NAAG, PCh, and s-Ins) shown in Figure 7.

## Discussion

Our simulation results show that ICA unmixes noise-free, multivariate data and extracts components closely resembling underlying spectra and that the ICA estimates closely track the ground truth-mixing coefficients. We also demonstrate that ICA offers superior consistency of results with simulated data compared to LCModel; while both results are nearly identical in the ideal case for LCModel, ICA is much more robust than LCModel in the nonideal case where the actual ground truth deviates from the assumed basis set, illustrating the effects of modeling inaccuracies. A close look at the effects of spectral correlations of the two sets of basis spectra reveals that the varying degrees of correlations of LCModel estimates in the nonideal case are due to the extent of the differences of spectral patterns between the models.

A wealth of information can be gleaned from the ICA results alone, by closely examining ICA's performance in extracting modeled resonances having different statistical properties. The illustration in Figure 4, where the modeled resonances of m-Ins and Gly are compared with their matching ICs, helps bring out the limitations and advantages of the ICA approach. The modeled spectra are correlated to each other, due to their common peak at ∼3.56 ppm. However, as the variability associated with that peak does not covary with other peaks in the modeled m-Ins resonance, ICA resolves the peak at 3.56 ppm separately and thus provides a clean estimate of Gly. As ICA minimizes mutual information among the components, the 3.56-ppm peak does not appear in the m-Ins like component, even though modeled spectrum has a 3.56-ppm signal, thus clearly exposing the limitation of ICA in extracting such resonances. Nevertheless, the extracted component substantially resembles m-Ins and, moreover, provides highly accurate estimates of m-Ins. So, rather than a limitation, it is an opportunity that ICA provides to extract resonances with singlet peaks, even in the presence of spectrally colocated strong resonances.

At the same time, resonances with multiple peaks that tend to be correlated with other (modeled) resonances, are not likely strictly independent to begin with, and therefore are difficult to resolve exactly using ICA, as evident from the slightly lower spectral correlations of such resonances (Table 1). However, even the lowest spectral correlation (other than m-Ins), that of Glc due to strong overlaps with Tau (*r* ∼0.41), is at ∼0.95. The low spectral correlations do not necessarily hurt ICA estimation, especially when the resonances are strong, for an error in their estimation is acutely felt.

Our in vivo results demonstrate that ICA can resolve signals of interest from the confounding artifacts and can group covarying resonances together. The estimates of identified components resembling Cr, NAA, PCh, and m-Ins signals, while including other covarying resonances (Fig. 7), nonetheless demonstrated strong correlations with the LCModel estimates of the identified metabolites. The weak correlation involving NAAG may be attributable to LCModel's limitation in resolving NAAG from NAA; though it makes sense to present NAA + NAAG for real data, we could not present that as our estimates are NAA normalized. An ICA component associated with the s-Ins signal is also consistently extracted by ICA, perhaps due to the lack of overlap with any other signal. Elevated s-Ins in the current data set may be due to effects of alcohol abuse (Viola et al. 2004) or aging (Kaiser et al. 2005).

The ICs that are unidentified include baseline and broadening components and resonances of interest, such as those from Asp, Glu, Gln, and GABA, indiscernible from such confounds. We acknowledge the difficulty in discerning resonances with multiple peaks, such as those from Glu + Gln, from the in vivo data, which LCModel estimates with reasonable accuracy. In our future study, we will provide modifications to ICA, by incorporating prior information, in the form of constraints in the ICA algorithm (Lin et al. 2010) to improve the estimations of such metabolites. Appropriate preprocessing steps to effectively reduce noise or baseline artifacts may also improve ICA's estimation accuracy, as our simulations indicate. Finally, the ICA approach may benefit from the use of all available complex time-domain data, rather than just the real part of the data that we used in this study, with very good performance. These strategies to improve ICA performance will also be explored in the future study.

Clearly ICA, which cannot analyze spectra individually, cannot replace the curve-fitting methods, such as LCModel, in individual spectral analysis. However, ICA can be very useful in the analysis of multiple spectra, and possibly offer a systemic advantage in such cases, as it makes full use of the information by collectively analyzing “complete” spectra, rather than just the quantified estimates. The ICA approach described in this report can be potentially applied to spectra from multiple voxels in a spectroscopic imaging data set from one or more subjects. It can also be applied to analyze data from two different study populations or tissue types and discriminate one group from the other. Conceivably, ICA can also play a complimentary role to model-based methods by identifying hidden structures underlying the data and help choose better “model”.

## Conclusion

We systematically compare the performances of ICA and LCModel in analyzing MR spectra and demonstrate, using noise- and artifacts-free simulations, that the data-driven ICA approach is more robust to variations in the spectral profiles underlying the data. Further, we show that composite spectra can be resolved to extract components substantially resembling modeled metabolite resonances, using independence criteria alone and that ICA can extract components from simple singlet signals, such as Gly, that overlap with other signals. We discuss the limitations and advantages of ICA in spectral decomposition in detail, and show that the ICA estimates, which exhibit a highly linear relationship with ground truth, can be very useful in analyzing a group of spectra. Furthermore, we apply ICA to analyze in vivo ^1^H-MRS spectra and show that ICA can extract components associated with NAA, Cho, Cr, and m-Ins in the presence of confounding artifacts. Finally, we show that ICA can be very useful, in extracting certain weak metabolites with singlet resonances, such as s-Ins and can provide visibility of resonances that covary. Together, these results suggest that ICA could be useful for collective analysis of multiple MR spectra.
